# Multifaceted Control of GR Signaling and Its Impact on Hepatic Transcriptional Networks and Metabolism

**DOI:** 10.3389/fendo.2020.572981

**Published:** 2020-10-08

**Authors:** Stine M. Præstholm, Catarina M. Correia, Lars Grøntved

**Affiliations:** Department of Biochemistry and Molecular Biology, University of Southern Denmark, Odense, Denmark

**Keywords:** Glucocorticoid receptor, chromatin, transcription, metabolism, liver

## Abstract

Glucocorticoids (GCs) and the glucocorticoid receptor (GR) are important regulators of development, inflammation, stress response and metabolism, demonstrated in various diseases including Addison's disease, Cushing's syndrome and by the many side effects of prolonged clinical administration of GCs. These conditions include severe metabolic challenges in key metabolic organs like the liver. In the liver, GR is known to regulate the transcription of key enzymes in glucose and lipid metabolism and contribute to the regulation of circadian-expressed genes. Insights to the modes of GR regulation and the underlying functional mechanisms are key for understanding diseases and for the development of improved clinical uses of GCs. The activity and function of GR is regulated at numerous levels including ligand availability, interaction with heat shock protein (HSP) complexes, expression of GR isoforms and posttranslational modifications. Moreover, recent genomics studies show functional interaction with multiple transcription factors (TF) and coregulators in complex transcriptional networks controlling cell type-specific gene expression by GCs. In this review we describe the different regulatory steps important for GR activity and discuss how different TF interaction partners of GR selectively control hepatic gene transcription and metabolism.

## Introduction

Any living organism must adapt and respond to the surrounding environment to maintain its existence. For multicellular organisms such as mammals, this includes daily transitions between different physiological conditions including sleep/awake, fasted/fed, and physical inactivity/activity. Moreover, occasional response to environmental changes such as confinement, predator stress, extreme temperatures, inflammation and prolonged lack of food is critical for survival. Glucocorticoids (GCs) serve as important endocrine signaling molecules controlling many molecular signaling pathways that enable cells in the organism to respond to different extrinsic cues. This is particularly evident for cellular responses in the arousal state including the transitions mentioned above. Importantly, pathophysiological conditions leading to dysfunctional GC signaling have dramatic effects on many important biological functions including development, inflammatory response, reproduction, cognitive function, anxiety, circadian entrainment, cardiovascular regulation and cellular metabolism in a tissue-specific manner (1). For example, uncontrolled GC secretion observed in Cushing's syndrome leads to metabolic complications such as type 2 diabetes and osteoporosis, which are also observed in situations of prolonged treatment with GCs. In contrast, conditions of low GC production, seen in Addison's disease, are associated with muscle weakness, low blood pressure and weight loss (2).

**Figure 1:**
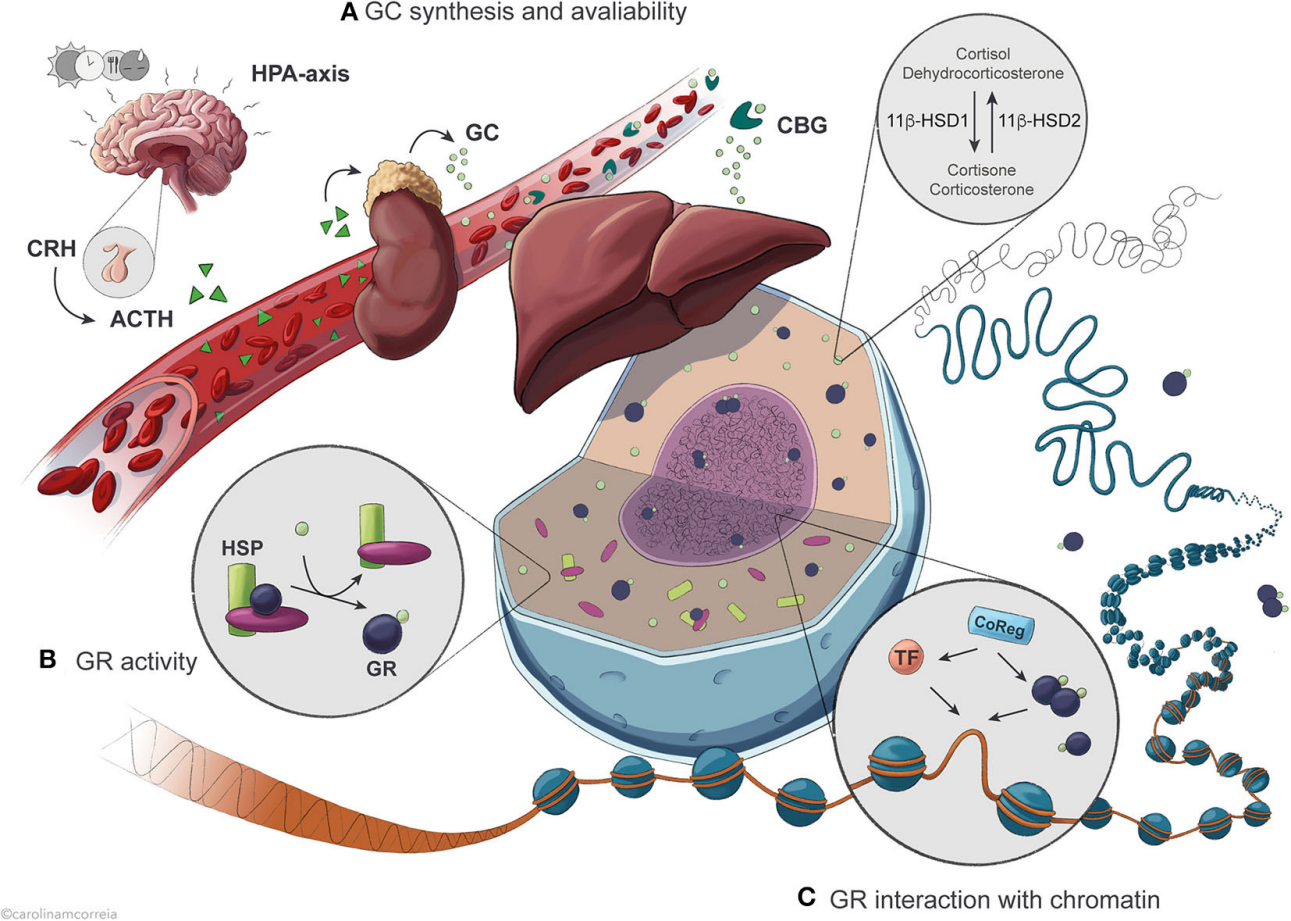
Overview of the regulatory levels affecting GR activity in the control of hepatic transcription. **(A)** Circadian and ultradian synthesis of GCs is controlled by the HPA axis in response to external stimuli including feeding, stress, light and circadian timekeepers. Availability of active GCs is further influenced by binding to the serum protein CBG and by intracellular conversion catalyzed by the enzyme 11β-HSD1/2. **(B)** Once in the cell, GCs are bound by the GR with an affinity that is conditioned by association with chaperone complexes containing HSPs, expression of specific GR isoforms and GR protein turnover. **(C)** GR exerts its action after translocation to the nucleus, where it binds GRE sequences in the DNA to regulate transcription of target genes as a result of dynamic interaction with different TFs and coregulators.

Glucocorticoids exert their actions primarily by binding to the glucocorticoid receptor (GR or Nr3c1), which is expressed in most cells in mammals. Yet, GCs have highly tissue/cell-specific effects regulated by multiple mechanisms. As a DNA-binding transcription factor (TF), GR is primarily involved in the control of gene expression, with transcription of GR target genes in a given cell being controlled by three overall mechanisms (Figure 1). First, activity of GR is directly correlated with the amount of GC molecules available in the cell. This is controlled by adrenal GC synthesis and local availability of GCs in the cell. Second, expression of active GR in the nucleus determines the molecular response to GCs. This is regulated by GR turnover (synthesis and breakdown), expression of different GR isoforms, posttranslational modifications (PTMs) and nuclear translocation. Third, genomic action of GR is controlled by cell type-specific accessibility of GR response elements (GRE) in the genome in synergy with cell-specific TFs, coregulators and regulatory RNAs. In this review we will discuss all three regulatory aspects of GR signaling with a specific focus on GR interaction with the genome. We will primarily refer to studies from mouse liver tissue to discuss recent insights to hepatic gene regulatory networks and metabolism controlled by GCs. This will specifically be related to the hepatic transcriptional response to the circadian rhythm, feeding and fasting.

## Regulation of Glucocorticoid Secretion and Availability in the Cell

Glucocorticoids (cortisol in humans; corticosterone in rodents) are steroid hormones secreted circadianly by the adrenal cortex. Their daily levels peak immediately before the active phase (early morning for humans; early evening for rodents) in anticipation of a waking state, but also in quick response to external stimuli such as stress, hypoglycemia and exercise (3, 4). The hypothalamic-pituitary-adrenal (HPA) axis controls and maintains GC secretion into the bloodstream (Figure 1A). The hypothalamus produces corticosteroid-releasing hormone (CRH), stimulating the pituitary gland to secrete adrenocorticotropic hormone (ACTH), which in turn promotes GC secretion by the adrenal gland (5). As many other hormones, including growth hormone and insulin (6, 7), GCs are secreted in an ultradian pattern with pulsatile secretion once every 60 to 90 min, as a result of feedback and feedforward mechanisms between ACTH, CRH and GC secretion keeping GC levels in a physiological range (4, 8, 9). The circadian secretion of GCs results partly from oscillations in ACTH secretion, but mostly from varying adrenal sensitivity to ACTH (3–5). In the blood, GCs circulate in association with corticosteroid-binding globulin (CBG) or, to a lower extent, albumin, and only a small fraction remains unbound in most vertebrates. As only free GCs diffuse into the target cells, CBG modulates GC bioavailability (10–12). Disruption of CBG expression in mice leads to reduced total serum GC (13) and as CBG and albumin are synthesized by the liver, it is possible that hepatic regulation of GC-binding proteins modulates the levels of available GC.

Additionally, non-adrenal production of cortisol has been described in visceral adipose tissue and liver via the conversion of inert cortisone catalyzed by the enzyme 11β-hydroxysteroid dehydrogenase type 1 (11β-HSD1) in humans (dehydrocorticosterone to corticosterone in rodents), and reversely by 11β-HSD2 (14, 15) (Figure 1A). Liver activity of this enzyme is particularly relevant to the whole-body non-adrenal production of cortisol; however, HPA axis feedback mechanisms likely blunt any systemic effects (15). Therefore, activity of 11β-HSD1 mostly contributes to locally maintaining intracellular levels of active GCs in the liver and visceral adipose tissue, fine-tuning the highly variable GC levels. This enzyme thus regulates the availability of receptor-active GCs in the cell, modulating access to GR and amplifying GC effects (16–18). In mice, absence of 11β-HSD1 leads to an inability to produce active GCs from the inert form, resulting in compensatory activation of the HPA axis, increased basal corticosterone levels and failure to fully elicit a hepatic gluconeogenic response to fasting, similarly to absence or impairment of GR (19). Dysregulation of 11β-HSD1 expression and activity is associated with apparent hypercortisolemia, disrupted metabolism and HPA axis function, obesity, type 2 diabetes and metabolic syndrome; however, the specific contribution of the enzyme to these processes is still controversial (16, 18).

## Circadian Control of Glucocorticoid Levels

The circadian synthesis and secretion of GCs by the adrenal glands is controlled by both the local molecular clock and the central clock in the suprachiasmatic nucleus (SCN) via a sympathetic neuronal pathway, and can be blunted by stress stimuli (3, 4). The SCN is important for GC rhythmicity, as it regulates the hypothalamic-hypophysial portions of the HPA axis affecting CRH secretion (20–22). During light-induced HPA axis-independent GC secretion, the SCN directly activates the adrenal glands via the adrenal sympathetic nerves, suggesting that GCs can act as SCN-gated mediators of the light stimuli to entrain metabolic-responsive peripheral clocks (5). The ubiquity of GR expression and the marked circadian secretion of GCs imply that these are efficient SCN-driven synchronizers of peripheral clocks and, specifically in the liver, are fundamental for the circadian expression of metabolic genes, even with contribution from other hormonal signals and entrainment factors (3, 4, 23). However, GCs do not affect the central clock, since GR is not expressed in the SCN (3, 23).

Unlike the SCN, the phase of peripheral clocks can be modulated by feeding, and even uncoupled from the SCN (3). As a metabolic organ, the liver is particularly responsive to feeding patterns, which can lead to desynchronization of its peripheral clock from the central clock (24, 25), an entrainment partly mediated by GCs (26–29). The interplay between eating behavior and GCs can be observed during day-restricted feeding of mice (opposite to their normal feeding pattern), leading to secretion of GCs with two distinct peaks instead of a single one, with one being feeding-responsive (before feeding time, in the early morning) and the other light-entrained (before the normal active period, in the early evening) (3, 4, 27, 30). Misalignment also occurs as a result of the disruption of normal activity patterns due to jet lag, shift work, sleep disorders or social jet lag, and associates with the development of metabolic disorders, such as diet-induced obesity and non-alcoholic fatty liver disease (31).

## GR Structure, Splice Variants and PTMs in the Modulation of GR Activity

The effects of GCs are mediated by GR through its three functional domains: a hydrophobic C-terminal ligand-binding domain (LBD) containing a ligand-dependent trans-activation portion (τ_2_, or AF2), a zinc-finger DNA-binding domain (DBD) located adjacently, and an N-terminal trans-activation domain (τ_1_, or AF1) (32–34). There is extensive alternative splicing and translation of human GR, impacting cell-specific GC actions. Alternative splicing originates multiple isoforms varying primarily in the DBD and the C-terminal LBD/AF2, while multiple translational start sites give rise to GR proteins with different lengths of the AF1 domain. The expression of some GR isoforms is evolutionarily conserved, but while many have shown biological relevance in humans (35), isoforms in rodents are less characterized. In humans and rodents, GRα (referred to simply as GR henceforth) is considered the canonical GR isoform that mediates most actions of GCs and is the primary isoform expressed in most tissues. Alternative splicing of the GR primary transcript in humans and rodents can give rise to additional GR isoforms, including GRβ, which has a truncated C-terminus, resulting in an inactive AF2, with compromised ability to bind GCs. Thus, GRβ is considered dominant negative (36, 37). Although expressed to a lower level than GRα, GRβ is considered a functional TF in a number of tissues, including the liver (36, 38). Additional isoforms include the widely expressed GRγ, which exhibits similar affinities to both GCs and DNA as GRα, but has a compromised transactivation potential and is associated with GC resistance. Expression of GR is also affected by the activity of miRNA molecules that bind to the 3′ UTR of GR transcripts, affecting their stability and preventing their translation (37). Additionally, lncRNAs such as Gas5 repress ligand-activated GR activity by binding to its DBD as a decoy GRE in starvation conditions, leading to suppression of GC-stimulated mRNA expression of key gluconeogenic enzymes *G6Pase* and *Pck1* during fasting (39).

In addition to the coregulatory function of specific GR isoforms, the activity of hormone-bound GR in different tissues can be modulated by specific sets of PTMs (40). For example, upon hormone binding, ligand-selective phosphorylation of the GR affects GR-mediated transcriptional activity and recruitment of coregulators, and is thus involved in directing and modulating GR action as a repressor or activator, namely via crosstalk from other signaling pathways such as in GSK3β-mediated phosphorylation (40–45). The relevance of PTMs on the GR protein and their effects on GR function are also illustrated by the protein-protein interactions between clock components and GR leading to suppression of GR activity via acetylation of a lysine residue by the CLOCK protein, potentiated by the presence of BMAL1 (46). Additionally, modifications such as GC-dependent phosphorylation reduce GR stability and half-life by tagging it for ubiquitination and subsequent degradation, and also influence its subcellular localization (37, 43, 44, 47–49). Other PTMs affecting GR function include SUMOylation, which reduces protein stability and regulates transcriptional activity, as well as nitrosylation and oxidation, both associated with reduction of GC-binding (37).

## Regulation of GR Translocation to the Nucleus

Inactive GR is located in the cytoplasm, monomerically associated with a multimeric chaperone complex important for GR stability, folding and translocation (Figure 1B). The maturation of the complex involves a stepwise ATP-dependent assembly from the initial GR-HSP70-HSP40 complex, to the recruitment of HSP90 and Hop facilitating the assembly of a final high GC affinity complex consisting of GR, HSP90, p23, and FKBP51 (50). Circulating GCs enter the cells via diffusion across the cell membrane and interact with GR. Upon ligand-binding, a FKBP51-FKBP52 switch exposes the GR nuclear localization signals, which are recognized by importins and nucleoporins, facilitating the translocation of activated GR through a nuclear pore via microtubules (50, 51). Disruption of FKBP52 leads to reduced expression of GR target genes in the liver and augmented hepatic steatosis as a result of diet-induced obesity (52), also observed in liver-specific GR knock out (L-GRKO) mice (26), demonstrating a functional role of the multimeric chaperone complex for hepatic GR function. In general, the subcellular location of GR follows the diurnal GC concentration (53). However, both ligand-bound and unbound GR shuttle dynamically between the nucleus and the cytoplasm with a variable rate, consequently regulating GR activity. Aberrantly high cytosolic pH and chemical stress can lead to dissociation of HSP90 and increased nuclear import of GR. GR nuclear translocation can also be regulated by context-specific PTMs, e.g., phosphorylation of GR by kinases like MAPKs, CDK, and GSK3 (50). In the liver, factors including HDAC6 and REV-ERBα have been found to affect GR translocation, thus affecting GR activity (53, 54).

## Genomic Actions of GR: General Concepts

**Figure 2:**
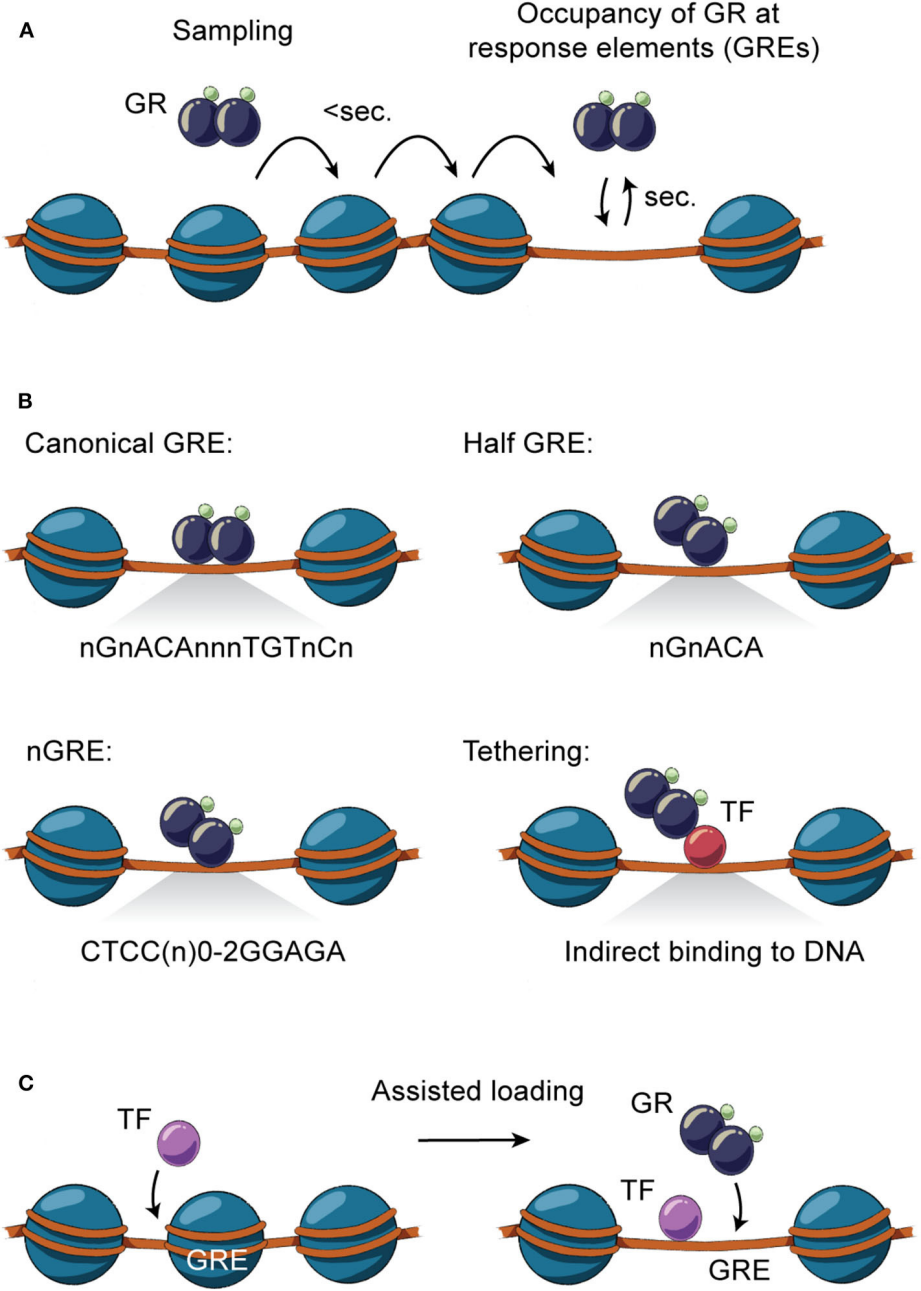
Direct and indirect GR-DNA interactions. **(A)** GR interacts dynamically with DNA. Freely diffusing GR occupies chromatin with residence time in milliseconds, whereas GR binding at specific regions of chromatin is measured in the order of seconds. **(B)** GR interacts directly with DNA by binding to canonical GRE (nGnACAnnnTGTnCn), half-sites (nGnACA) and nGRE (CTCC(n)_0−2_GGAGA) or indirectly by tethering to DNA-bound TFs by protein-protein interactions. **(C)** TFs can assist the loading of GR, or vice versa, by facilitating an accessible chromatin environment at the regulatory site.

Following nuclear translocation, GR accumulates at specific gene regulatory regions (e.g., enhancers) depending on the DNA sequence, occupancy of other TFs, organization of nucleosomes and higher order chromatin structures (Figure 1C). GR residence time at specific regions of chromatin lasts seconds, whereas freely diffusing unbound GR occupies chromatin in milliseconds (55) (Figure 2A). This enables GR to efficiently probe tens of thousands of putative enhancers within a short time frame and initiate transcription of hundreds of genes within minutes of activation by hormone (56). Also, the dynamic nature of chromatin interaction is shared by transcriptional coregulators known to interact with GR (57), both likely playing an important kinetic role in GC-regulated gene expression, including the duration and frequency of transcriptional bursting (58). As a result of the pulsatile secretion pattern, GC concentration in the serum is highly dynamic, allowing a rapid transcriptional response that can be translated into a fast biochemical response (59). For example, transcriptional bursting has been linked to a fast-acting metabolic switch in hepatic glucose metabolism, where expression of gluconeogenic genes such as *G6pc* and *Pck1* is rapidly decreased in response to feeding (60), the latter being regulated by GCs (61, 62).

### Direct and Indirect GR Interaction With the DNA Template

Genomic occupancy of GR is facilitated by direct GR binding to GREs on DNA as a monomer, homodimer or tetramer (63) (Figure 2B), with the tetrameric structure being suggested as the final active form of GR (64). GR binds directly to the canonical DNA motif consisting of inverted repeats separated by 3 bps (nGnACAnnnTGTnCn) or to half-sites of these inverted repeats (nGnACA) (63, 65) and degenerate versions of these (66). In addition, GR can bind other inverted repeats separated by 0-2 bps (CTCC(n)_0−2_GGAGA) (67, 68), termed negative GREs (nGRE). Besides binding directly to DNA, GR can occupy enhancers by tethering to DNA-bound TFs by protein-protein interactions (63, 65).

Binding of GR to canonical DNA motifs as homodimers and tetramers is generally associated with GC-mediated transactivation (63, 69–71). Also, studies suggest that GR association with GR half-sites is linked to active gene expression (63, 65). Once GR is associated with enhancers, GC-induced transactivation involves recruitment of transcriptional coactivators to facilitate chromatin remodeling, histone hyperacetylation and mediator recruitment which leads to recruitment and/or increased activity of RNA polymerase II at juxtaposed gene promoters (56, 72–74). In contrast, GC-mediated transrepression has been widely discussed, and hence several different models have been presented, including direct binding of GR to nGRE motifs, interaction with DNA sequences bound by other TFs, tethered GR binding to transactivating TFs, redistribution of monomeric GR, sequestering of transactivating coregulators and/or GR-regulated expression of negative modulators of transcription (75). Even though nGREs have been associated with transcriptional repression (67, 68, 76), their role has been debated (63, 72, 74). For example, recent studies found no enrichment of nGREs at enhancers juxtaposed to GC-repressed genes (74). In contrast to enhancers induced by GC, repressed enhancers show marginal canonical GR binding motifs, suggesting that GR binds other DNA motifs (77) or tethers to other TFs (78). This type of GR interaction with DNA is generally believed to be mediated by monomeric GR, based on structural studies of the GR DBD and mice expressing a mutant GR (GR^dim^) unable to achieve DBD dimerization (69, 76–79). Although mice expressing GR^dim^ indeed show reduced GR transactivation ability in the liver and maintain transrepressive activity (70), studies have suggested that GR^dim^ forms dimers in the nucleus through another dimerization surface of the LBD (80). This suggests that binding to GR half-sites or other DNA motifs may be mediated by GR dimers, where possibly only one part of the dimer binds directly to DNA (Figure 2B). Cistromic analysis of GR and GR^dim^ in the liver and in macrophages suggests extensive GR binding to chromatin through GR half-sites, which in many cases colocalizes with lineage-determining TFs driving cell-specific gene transcription (63, 70). Accordingly, GC treatment has been suggested to induce pronounced GR redistribution from GR half-sites to canonical GREs leading to reduced transcription of genes controlled by lineage-specific TFs (63). Introducing a mutation that completely disrupts direct GR binding to DNA (GR^Δ*Zn*^) leads to a perinatal lethal phenotype similar to knock out of GR, emphasizing an essential function of direct binding to DNA. Interestingly, studies of mouse embryonic fibroblasts isolated from GR^Δ*Zn*^ mice show that direct GR-DNA interaction is essential for both transcriptional activation and repression by GCs, arguing that tethering is not a dominant mechanism for GR transrepression (81). Thus, genomic action of GR is primarily mediated by multimeric or monomeric actions involving direct interaction with the DNA template.

### GR Interaction With Chromatin

GR binding to DNA is not solely dependent on the DNA sequence of the GRE. As GR binding sites are part of enhancer regions organized in higher order chromatin structures, occupancy of GR to specific regions of the genome is determined by a number of interdependent factors. This includes selective chromatin accessibility, epigenetic modifications of the histones, and the presence of other signal-dependent TFs, lineage-determining TFs and transcriptional coregulators (56). In the mouse liver, GR binds at least 11,000 distinct regions which are primarily located in intronic and intergenic distal regions (26, 61, 63, 72, 82). The vast majority of the GR binding sites are accessible prior to GC stimulation (pre-accessible chromatin) and only some are *de novo* remodeled following GR recruitment (72). Similar findings are observed for other cell types (56, 83, 84), demonstrating that selective GR occupancy of chromatin is largely determined by the accessibility of GREs. This pre-programmed chromatin landscape is shaped by cell-specific TFs and interacting coregulators that facilitate an accessible chromatin environment thereby assisting the loading of other TFs to the chromatin (discussed below; Figure 2C) (85). Accordingly, when comparing the liver cistrome across a number of well-described GC-responsive cell types, more than 80% of GR binding sites are unique to the liver and only 0.5% of the binding sites in the liver are shared with other cell types (72). This correlates with the findings that GR-occupied enhancers active in one cell type are inaccessible and nucleosomal in another cell type (73). GR has also been found to facilitate binding of other TFs to enhancers in the liver by establishment of accessible chromatin (72). In fact, binding of GR to genomic regions with different levels of chromatin accessibility has been linked to the type and strength of the GRE motif, with weaker motifs being found at nucleosome-depleted enhancers, compared to more nucleosomal dense sites (73).

### Control of Gene Transcription by Recruitment of Coregulators and Chromatin Remodeling

Upon GR binding to chromatin, the local nucleosome-sparse region expands and the accessibility of the chromatin is further increased trough recruitment of chromatin remodeling complexes such as SWI/SNF and additional TFs (86–88). In addition, GR facilitates recruitment of widely expressed coactivators including histone acetyl transferases CBP, P300, GRIP1, PCAF and SRC-2 and components of the Mediator complex such as MED1 and MED14 (56, 66, 73, 89, 90). Moreover, other important GR coactivators have been identified in the liver, including CRTC2 (91), SIRT1, PGC-1α (92), ASCOM complex (93) and SETDB2 (94). On the other hand, GR has been found to interact with corepressors including SMRT (95), HDAC1 (96), CtBP (97), SMAD6-HDAC3 (98), CRY1 (99) and recently TAZ (100), although these interactions are not necessarily associated with transcriptional repression. The wide variety of coregulator interactions allows transcriptional fine-tuning of specific genes in a given cell in a concerted response to cellular signals and circulating GC levels.

Local recruitment of GR and associated coregulators to specific enhancers is translated to a transactivation potential by assembly into higher order enhancer-enhancer and enhancer-promoter condensates (101), facilitating localized increased concentration of the transcriptional machinery (102). Interestingly, interaction between promoters and enhancers occupied by GR is mostly established prior to GC stimulation (103, 104), suggesting that GC treatment does not necessarily lead to new chromosomal interactions but rather increases existing interactions between GR-occupied enhancers and GC-regulated target genes (74). Importantly, availability of GCs has been shown to be central for this differential interaction, suggesting that rapid regulation of gene transcription in response to changes in GC levels not only involves dynamic loading of GR and coregulators on the genome but also differential regulation of enhancer-promoter interaction (103).

## GR Operates in Transcriptional Networks to Control Hepatic Gene Expression

**Figure 3:**
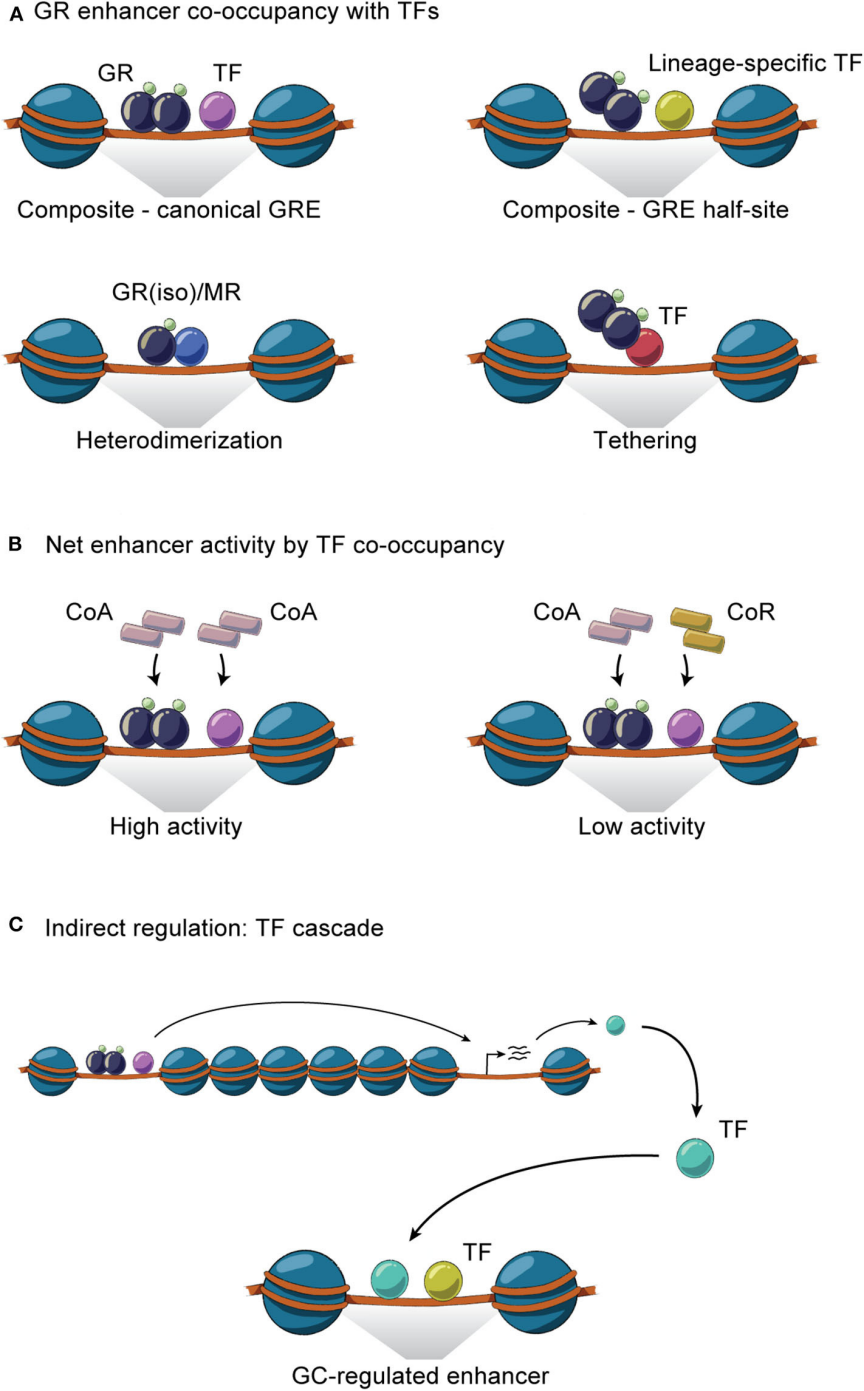
GR interaction with TFs on chromatin. **(A)** GR and TFs co-occupy enhancers through homodimeric or monomeric GR binding together with TFs at composite sites, by heterodimerization and through tethering. **(B)** GR- and TF-mediated recruitment of coactivators (CoA) and/or corepressors (CoR) to co-occupied regulatory sites controls the net enhancer activity. **(C)** Indirect GR-TF interaction involves TF cascades, where the expression of GR regulates the expression of TF or vice versa.

The general GR working model described above illustrates that cell-specific GR actions are orchestrated by auxiliary lineage-determining and signal-dependent TFs. As any given cell expresses multiple cell-specific TFs that shape the accessible chromatin landscape, it is evident that GR-GC action in a given cell is controlled by signaling pathways regulating the activity and expression of these TFs. For example, the liver receives a variety of context-dependent signals controlling specific signaling pathways including circadian cues, insulin, glucagon, growth hormone and free fatty acids, that collectively shape and are shaped by the GC response in hepatocytes. These different signals are integrated in spatial and temporal TF signaling networks that regulate and fine-tune the hepatic transcriptional response. GR interaction with different TFs and the importance of these interactions for transcriptional regulation have been investigated for decades (105). Recently, several key genome-wide studies in mouse liver tissue have demonstrated that GR interacts with a large repertoire of TFs and that these interactions are diverse, bidirectional, dynamic and highly context- and cell-specific (Table 1). The interactions between GR and TFs can be classified as direct or indirect. Direct interactions cover protein-protein interactions or concurrent and co-localized binding to regulatory sites in the chromatin (Figure 3A), impacting coregulator recruitment, and consequently enhancer activity (Figure 3B). Indirect interactions involve TF cascades, where the expression of one TF regulates the expression of another TF (Figure 3C).

**Table 1:** Examples of hepatocyte expressed transcription factors interacting with GR on chromatin.

**Transcription factor**		**Signals regulating TF activity**	**Interactions with GR**	**Model**	**References**
**Metabolism**					
C/EBPα	CCAAT enhancer binding protein alpha		Co-localization	Mouse liver	(72)
C/EBPβ	CCAAT enhancer binding protein beta		Co-localization. C/EBPβ-mediated assisted loading of GR	Mouse liver	(63, 72)
COUP-TFII	Orphan nuclear receptor chicken ovalbumin upstream promoter-transcription factor II	9-cis-retinoic acid All-trans-retinoic acid	Protein-protein interaction. Co-localization on chromatin	H4IIE and HepG2	(106)
CREB1	CAMP responsive element binding protein 1	Glucagon	GR-mediated assisted loading. Co-localized binding	Mouse liver	(61, 107–109)
E47			Co-localization on chromatin. E47 is important for GR recruitment.	Mouse liver	(110)
FOXA	Forkhead box A1		Half-site tethering	Mouse liver	(63)
FOXA2	Forkhead box A2		FOXA2-mediated assisted loading of GR. Co-localization at site	Mouse liver and primary mouse hepatocytes	(63, 109)
FOXO1	Forkhead box O1	Insulin	Co-localization on chromatin and protein-protein interaction	Mouse liver H4IIE	(61, 110, 111)
LXRα	Liver X receptor alpha	Oxysterols	Competes with GR for binding at target sites	HepG2	(112)
LXRβ	Liver X receptor beta	Oxysterols	Facilitates GR binding to selected GREs	Mouse liver	(113)
HNF6	Hepatocyte nuclear factor 6		Half-site tethering	Mouse liver	(63)
PPARα	Peroxisome proliferator activated receptor alpha	Fatty acids, eicosanoids, phospholipids, polyphenols	Co-localization on chromatin	Primary mouse hepatocytes	(114)
**Circadian clock**					
BMAL1	Brain and muscle ARNT-like 1	Circadian	Protein-protein interaction. GR is tethered to BMAL1-CLOCK complex. Co-localization on chromatin	Mouse liver	(26, 115)
CLOCK	Circadian clock regulator	Circadian	Protein-protein interaction. GR is tethered to BMAL1-CLOCK complex. Co-localization on chromatin	Mouse liver	(26, 115)
CRY1/CRY2	Cryptochrome circadian regulator 1/2	Circadian	Co-localization on chromatin through tethering. Protein-protein interaction	HepG2 cells Mouse liver	(26, 99, 116)
PER1/2	Period circadian regulator 1/2	Circadian	Co-localization on chromatin	Mouse liver	(26)
REV-ERBα/β	Nuclear receptor subfamily 1 group D member 1/2	Circadian, hem	Protein-protein interaction. Co-binding to sites. REV-ERBα-mediated assisted loading of GR	Mouse liver	(26, 82)
RORα/γ	RAR related orphan receptor A/C	Circadian	Co-localization on chromatin	Mouse liver	(26)
**Development and growth**					
HNF1α	Hepatocyte nuclear factor 1 alpha		Co-localization at sites	Mouse liver PLC/PRF/5 cells	(117, 118)
HNF4α	Hepatocyte nuclear factor 4 alpha	Linoleic acid	Co-localization at sites	Mouse liver	(63, 117)
STAT5	Signal transducer and activator of transcription 5	Growth hormone. Cytokines	Protein-protein interaction. Co-localization at sites. STAT5 tethers GR to sites. STAT5 induces GR recruitment to sites	Mouse liver	(26, 119, 120)
**General**					
HSP90	Heat shock protein 90		GC-dependent co-localization on chromatin	Rat hepatoma HTC cells	(121)
p23	Prostaglandin E Synthase 3	Prostaglandin E Synthase 3	GC-dependent co-localization on chromatin	Rat hepatoma HTC cells	(121)

### Composite TF Interactions and Assisted Loading

At composite sites, GR binds to GREs and can functionally interact with other TFs bound to a neighboring site in the same regulatory region, co-operatively regulating enhancer activity. These binding sites can be overlapping or closely located on the DNA strand and involve GREs, half GREs and/or nGREs (Figure 3A). Many liver-expressed TFs have been found or suggested to co-occupy GR binding sites (Table 1). ChIP-seq experiments have confirmed the composite binding of CREB1, FOXO1, FOXA, HNF4α, HNF6, C/EBPα, C/EBPβ, PPARα, E47, STAT5, and REV-ERBα at several GR-occupied enhancers (26, 61, 63, 72, 82, 107, 108, 110, 114). In the liver, ChIP-seq data suggests that GR binds GRE half-sites together with lineage-determining TFs including HNF4α, C/EBPβ, HNF6, and FOXA (63, 72). In addition, AP-1 and SP1 motifs have been found to be enriched at GR binding sites (122) and the AhR binding site contains a GRE (123), suggesting that these TFs could work together with GR at specific sites to regulate transcription (124). However, further investigations are needed to determine the relevance of AP-1, SP1, and AhR on GR activity in the liver.

Several confirmed composite GR-TF interactions have been found to impact GR activity and hepatic metabolism, including C/EBPβ, E47, STAT5, and LXRβ, which are required for GR recruitment to specific sites (26, 72, 110, 113), in accordance with the model for assisted loading. For example, GR and E47 co-occupy many promoters and enhancers, working in synergy to regulate GC-induced metabolic genes. Studies using liver-specific E47 knock-out mice emphasize the importance of E47 in the recruitment of GR, FOXO1, and the mediator complex to composite sites. This cooperation affects glucose, fatty acid and lipid metabolism, which is demonstrated by E47 knock-out mice being protected from GC-induced hyperglycemia, dyslipidemia and hepatic steatosis (110). Another example is the C/EBP-facilitated assisted loading of GR. C/EBP has been found to occupy and prime the majority of GR target sites in the liver, making the chromatin accessible for GR binding. Disruption of C/EBP binding attenuates GR recruitment and GR-induced chromatin remodeling at composite sites (72). The concept of assisted loading is also found reversely, with GR assisting the loading of TFs including C/EBP and CREB1 at a subset of sites (Figure 2C) (72, 107). For example, GR-mediated assisted loading of CREB1 at a subset of CREB1 target enhancers doubles the number of CREB1 bound sites and increases chromatin accessibility, eventually leading to increased hepatic glucose production during fasting (107).

### Protein-Protein Interactions: Heterodimerization and Tethering at Chromatin

As mentioned above, multiple GR isoforms can be generated from the primary transcript and protein processing. Thus, GRα/β heterodimers can be formed on chromatin, impacting the activity of occupied enhancers (125–127) (Figure 3A). In fact, GRβ has been shown to have metabolic relevance in the liver. For example, feeding induces GRβ expression within 7 h, likely in response to insulin (36). This is supported by observations that hepatic GRβ expression increases in diet-induced obese mice (128). Overexpression of GRβ in mouse liver reduces expression of known GRα target genes such as *Pck1* and *Ppara*, associated with disrupted gluconeogenesis and increased hepatic lipid accumulation and inflammation, respectively (128, 129). Moreover, the GRβ-mediated increase in lipid accumulation is also seen in L-GRKO mice (26, 130), suggesting that GRβ may function as a negative regulator of GRα in hepatic fatty acid metabolism. Importantly, GRβ expression in a GRα-negative background leads to expression of a specific set of genes not regulated in the presence of GRα (129), suggesting that GRα and GRβ regulate each other's activities by mechanisms involving accessibility to chromatin, cooperation with TFs and coregulators and indirect regulation of enhancer activity (Figures 3A–C). Likewise, GR has been found to form a heterodimer with the mineralocorticoid receptor (MR) (34) in a number of different tissues and cells, including the hippocampus and mammary cells. Here, the GR-MR complex binds to GREs and regulates gene expression (131, 132). Although a GR-MR complex has not, to our knowledge, been shown to be functional in the liver, it has been suggested that GR-MR could regulate hepatic expression of *G6Pase* (133) (Figure 3B). However, further investigations are needed.

Besides heterodimerization on DNA, GR has been suggested to form other protein-protein interactions on chromatin which tether GR to enhancers independently of its DBD. This includes interaction with COUP-TFII, STAT5, PPARα and the molecular clock components BMAL1, CLOCK, and REV-ERBα, influencing GR activity and hepatic metabolism (82, 106, 115, 116, 119). For example, COUP-TFII protein interaction with GR is important for GC-induced promoter activity and hepatic *Pck1* gene expression (106). Also, GR is suggested to be recruited to a subset of sites via tethering to DNA-bound PPARα to regulate metabolic genes in the liver including *Pdk4* (114). Moreover, GR tethering to the BMAL1-CLOCK complex is suggested to repress hepatic *Rev-erb*α expression (115), demonstrating how GR and the molecular circadian clock interconnect to regulate shared gene programs.

### Controlling Enhancer Activity by Co-occupancy of Multiple TFs

The transcriptional effect of multiple TF interactions at enhancers can be evaluated by looking at the expression of juxtaposed target genes or at localized histone acetylation and mediator recruitment. In the case of TF cooperation at individual enhancers, activation of several TFs will result in synergistic effects on enhancer activity and gene expression. In contrast, TFs working independently at the shared enhancer would result in gene expression corresponding to a sum of the contribution from each TF. For example, composite GR-PPARα sites have been found to synergistically affect the expression of fatty acid oxidation and ketogenic genes while GR-CREB1 sites synergistically regulate gluconeogenic genes (107). Likewise, synergistic and additive regulation has been reported for genes controlled by GR and FOXO1 in co-occupancy (61). These cooperative effects likely reflect increased recruitment of coactivators to a given set of enhancers involved in transcription of a specific gene (Figure 3B).

In contrast to the synergistic action of composite GR-TF binding sites to increase enhancer activity, several studies have suggested negative regulation between GR and TFs occupied at a given enhancer. Such negative regulation can be understood as a competition between the TFs for a given DNA sequence. For example, in the liver, LXRα binds GREs together with its heterodimerization partner RXRα, thereby potentially competing with GR for binding to the same sites leading to differential regulation of genes involved in glucose metabolism (112). Another example is the GR isoform competition model, which seeks to explain how dominant negative GRβ functions as a negative regulator for GRα at some sites. Similarly, GR has been suggested to compete with AP1 at AP1 motifs with embedded GR half-sites (77). However, these competitional models do not agree with the dynamic nature of GR and most other TFs as these factors bind transiently to chromatin with residence times in a matter of seconds (55, 134), possibly allowing multiple factors to interact with the same site (135). Thus, GR-TF competition at composite sites is likely not a competition for the same response element. Instead, the negative regulation likely reflects the different coregulators recruited to the response element. Composite binding of different TFs recruiting coregulators of opposite activity or competition between limited amount of avaliable coregulators for binding to the specific TFs would balance the transcriptional response. For example, corepressors and coactivators have been suggested to bind GR in equilibrium, balancing GR activity (136), which has also been suggested for other nuclear receptors in the liver, including the thyroid hormone receptor (137).

### Regulating TF Networks by GR

The direct interaction between GR and other key TFs on chromatin in the liver can take different forms, as described above, to jointly regulate hepatic gene expression. However, indirect GR-TF interactions involving TF cascades are equally important, though more challenging to investigate, with several potential interaction steps (Figure 3C). Important indirect pathways have been studied in the liver. For example, GR binds GREs near core clock genes to induce transcription of *Per1, Bmal1, Cry1, Dbp* (138, 139). This in turn controls a range of circadian-regulated genes. In regards to energy metabolism, GR interacts with several key factors in TF cascades connecting and impacting different signaling pathways. For example, glucagon-mediated activation of CREB1 induces the transcription of YY1, which then induces the transcription of GR. This interaction cascade is important in hepatic gluconeogenesis (140). Moreover, GR induces the transcription of Klf9, which has been linked to the downstream induction of PGC1α expression and of hepatic gluconeogenic genes (141). GR interaction with PGC1α has furthermore been suggested to regulate mitochondrial oxidative phosphorylation (142). Additionally, GR induces the transcription of PPARα upon long-term fasting, initiating hepatic fatty acid oxidation and the ketogenic gene program (107).

## GR Regulatory Networks Impact Multiple Aspects of Hepatic Metabolism

**Figure 4:**
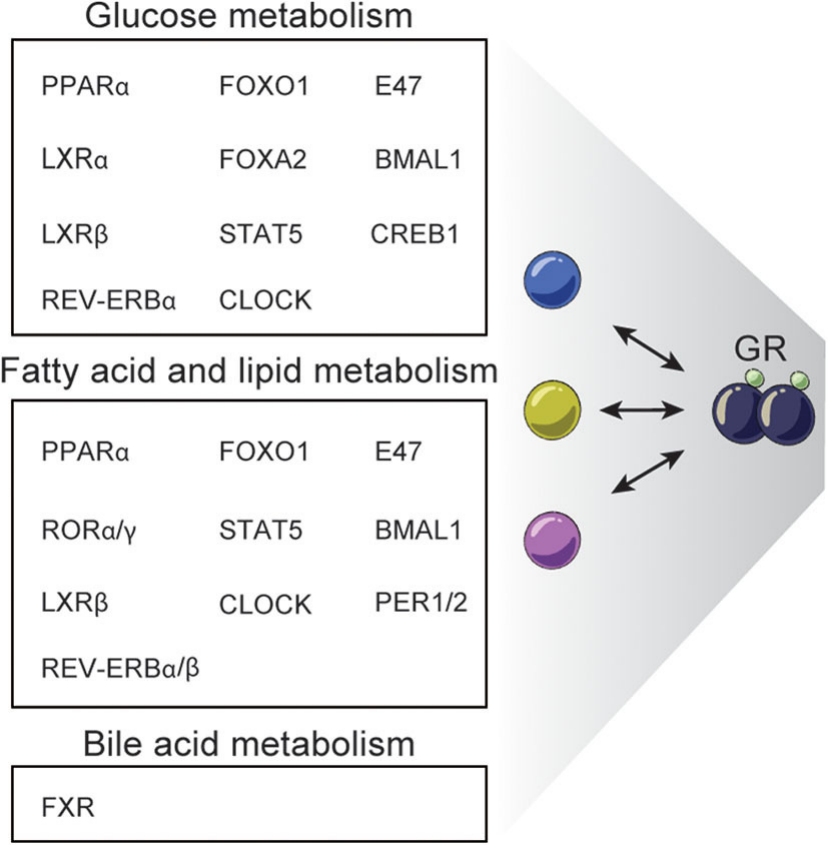
Examples of TFs interacting with GR regulating hepatic metabolism. GR interacts with different TFs to regulate specific processes in hepatic glucose, fatty acid, lipid, and bile acid metabolism.

The emerging studies in complex gene-regulatory networks controlled by GR and controlling GR activity emphasize the importance of the context-dependent action of GCs in tissues like the liver. Accordingly, genetic disruption of GR in the liver impacts a range of metabolic pathways leading to dysregulated glucagon synthesis, lipid metabolism, gluconeogenesis, urea metabolism and bile acid synthesis and uptake (26, 143–146). For example, L-GRKO mice and GR^dim^ mice show dysregulated glucose, fatty acid and bile acid metabolism (26, 144, 146). Reduced expression of key gluconeogenic genes including *Pck1, G6Pc*, and *Pfkfb3* in L-GRKO mice is linked to fasting hypoglycemia (26, 144–146), and around half of newborn albumin-alpha-fetoprotein-driven L-GRKO mice die within 48 h after birth, possibly due to hypoglycemia (120, 146). L-GRKO mice are more sensitive to insulin than WT littermates and liver glycogen content in L-GRKO mice is reduced (145). These effects of L-GRKO on glucose metabolism could in part be explained by the interaction with TFs such as CREB1, FOXO1, FOXA2, PPARα, E47, STAT5, LXRα, LXRβ, and circadian regulators (26, 61, 82, 107, 109, 110, 112–114) (Figure 4). Yet, the effects of L-GRKO on glucose metabolism seem to be partially compensated by increased gluconeogenesis in the kidney (145) and by a shifted hormonal balance involving reduced plasma concentration of insulin and increased glucagon levels, compared to WT mice (146).

Hepatic GR disruption also leads to decreased fat mass (145) and lower plasma triglyceride levels (26, 146), while free fatty acid plasma levels are similar in fasted and fed L-GRKO mice and WT mice (146). Recently, L-GRKO mice were reported to accumulate triglycerides in the liver and to develop hepatic steatosis (26), although this is controversial (130), but may be explained by the promoter controlling CRE expression. Many TFs have been found to work together with GR to regulate fatty acid and lipid metabolism including STAT5, PPARα, FOXO1, E47, LXRβ, CLOCK, REV-ERBα/β, CRY, BMAL1, RORα/γ, and PER1/2 (26, 82, 110, 111, 113, 114) (Figure 4).

Finally, disruption of hepatic GR function leads to dysregulated systemic bile acid homeostasis. Specifically, mice with hepatic GR knock down by shRNA have a reduced amount of bile acid in the gallbladder, elevated serum bile acid levels, impaired bile acid uptake/transport and are more susceptible to develop gallstones when fed on cholesterol-rich diet. Moreover, these mice do not undergo the normal changes in bile acid levels in the serum, liver and intestines in the fast-refeeding transition (144). GR^dim^ mice fed a lithogenic diet have elevated fasting serum bile acid levels and decreased gallbladder bile acid volume. These effects have been associated with interaction between GR and FXR, a key TF regulating bile acid metabolism (97, 144). GR deficiency reduces the expression of the classical FXR-target gene *Shp* encoding the SHP repressor, leading to increased expression of the rate-limiting enzymes in bile acid synthesis Cyp7a1 and Cyp8b1 (144). Additionally, dex-induced GR recruits the co-repressor CtBP to block FXR activity at shared sites related to bile acid gene metabolism, e.g. *Shp* promoter (97) (Figure 4).

## Examples of Key Hepatic Gene Regulatory Networks Controlled by GR

### GR Crosstalk With FOXO1

The daily change from the inactive fasting phase to the active feeding phase requires a major transcriptional reprogramming of the liver. This is particularly relevant at the transition between the unfed and fed states, which takes place around zeitgeber time (ZT) 12 (i.e., 6 p.m.) in nocturnal animals such as mice. The interaction between GR and the insulin-regulated TF FOXO1 is involved in driving this transcriptional transition. Pre-prandial high GC and low insulin levels are associated with GR and FOXO1 binding to chromatin, respectively, and regulation of target genes. In fact, in this fasted state, more than half of all FOXO1 binding sites are co-occupied with GR regulating gene expression. Conversely, the post-prandial increased insulin and reduced GC lead to reduced FOXO1 and GR occupancy, respectively, and reduced transcriptional regulatory activity. Importantly, more than 80% of feeding-repressed genes in the liver are associated with a nearby enhancer bound by GR, FOXO1 or both (61). One example of a metabolic gene coregulated by GR and FOXO1 in the liver is *Angptl4*, associated with the regulation of glucose and lipid metabolism. In a fasted state, GR and FOXO1 bind a specific GRE and forkhead box transcription factor response element (FRE), respectively, located in the regulatory region of *Angptl4*. GCs induce, while insulin abolishes, the occupancy of both factors at the region (111). Besides the direct interaction between GR and FOXO1 at enhancers in the liver, GR has been found to induce the expression of *Foxo1* gene in the liver and in this way indirectly regulate target genes (147). Furthermore, FOXO1 binding has been found at the promoter of GR, suggesting that the indirect interaction is bidirectional (148).

### GR Crosstalk With PPAR**α**

Like GR, PPARα is important for the hepatic response to fasting. The role of PPARα in regulating metabolism and inflammation as well as the importance of crosstalk between PPARα and other TFs, including GR, have been covered in detail in previous reviews (1, 149). The GR-PPARα interactions in the liver include co-localization to chromatin and coregulation of genes involved in lipid and glucose metabolism (150). More specifically, in co-ligand treatment of primary murine hepatocytes, 13% of GR peaks are co-bound with PPARα (114). Furthermore, other studies have found that, during fasting, GR and PPARα have a synergistic effect on genes involved in ketogenesis and fatty acid oxidation; however, the GR-PPARα interaction has been suggested to be indirect as GR induces the expression of PPARα and time-course experiments show a gradual effect of GR on PPARα activity (107).

### GR Crosstalk With STAT5

STAT5 is activated by the growth hormone through the growth hormone receptor-JAK2 signaling pathway and by cytokine signaling. In the liver, STAT5 is known to regulate genes involved in body growth, cell cycle, lipid, bile acid, drug and steroid metabolism (151). The STAT5 and GR signaling pathways are connected as exemplified by the reduced body size in mice with inactivated hepatic GR showing impaired growth hormone signaling (120). Furthermore, the importance of STAT5 and GR signaling is demonstrated in liver-specific STAT5 and STAT5 GR double mutant mice exhibiting hepatic steatosis and, for the double mutant, also hepatic carcinoma (130). The STAT5 and GR crosstalk at multiple levels. STAT5 and GR form protein-protein interactions in hepatocytes, which have been found to be important for postnatal growth and maturation-related gene expression. Mice expressing a point mutation in the GR DBD (GR^dim^ mice), previously suggested to reduce GR DNA-binding and GR dimerization (69, 79), have an unaltered ability to interact with STAT5 (120). These GR^dim^ mice have normal body size, suggesting that the joint GR-STAT5 regulation of growth genes happens through tethering of GR to the STAT5 bound sites or through half GREs in conjunction with STAT5 binding sites (119, 120). However, as mentioned above, more recent studies have found that GR^dim^ is able to dimerize and bind DNA (80), suggesting a reassessment of GR and STAT5 interaction type at shared sites.

Recently, it has been shown that high-fat diet feeding of mice leads to reprogramming of the hepatic GR cistrome primarily during the active feeding phase. Many sites with high-fat diet-induced increased GR recruitment are associated with increased STAT5 co-occupancy. These co-occupied sites showed increased enhancer activity and were associated with genes involved in fatty acid, lipid and glucose metabolism. Hepatocyte-specific STAT5 and GR KO mice demonstrated that STAT5 facilitated the recruitment of GR at gained sites, whereas GR had no effect on STAT5 recruitment. It is still unknown whether the increased STAT5 activity in obese mice is a response to altered growth hormone or cytokine signaling or if it originates from nutritional adaptations in the chromatin landscape (26).

### GR Crosstalk With Molecular Clock Components

In the liver, the effect of exogenous GCs on gene regulation is highly dependent on the time of administration. For example, in mice about eight times more genes are differentially regulated by GCs at daytime compared to nighttime. Pathway analysis shows a strong time-dependent regulation of genes in glucose and lipid metabolism (82), which has also been observed in studies looking at endogenous GC effects (26). Hence, timing of GC administration according to the endogenous GC levels has shown positive effects. Administration of GCs at ZT12, as opposed to ZT0, leads to less hepatic lipid accumulation and behavioral changes. This time-differential effect of GC is suggested to be caused by a disrupted circadian regulation of GC-target genes with administration at ZT0, which is supposedly more critical compared to an over-activation of GR at ZT12 (152).

This diurnal oscillation of GC action stems from cooperativity and multiple interactions between GR and the molecular clock components in the liver. For example, GR and central clock components including BMAL1, CLOCK, REV-ERBα/β, PER1, PER2, CRY1, CRY2, and RORα/γ co-occupy different genes involved in clock function and in metabolism (26, 82, 99). The cooperativity also involves different physical interactions between GR and clock factors on the chromatin level, regulating the expression of other clock factors and metabolic genes (see Table 1). For example, GR physically interacts with CRY1/2 in a GC-induced manner and, in the post-prandial phase, CRY1/2 represses GR activity on e.g. the expression of *Pck1*. CRY1/2 deficient mice have constitutively high GC levels and exhibit glucose intolerance, suggesting reduced suppression of HPA axis and increased GR activity in the liver (99).

It has been long known that GC and GC-activated GR influence the expression and circadian phase-shifts of several clock factors, including *Per1, Dbp* and *Cry1* (23, 30, 139). In fact, GR is recruited to the promoters and enhancers of all central clock genes, suggesting a gene regulatory function of GR (26). Reversely, molecular clock elements also affect GR function, as exemplified by the previously mentioned binding of REV-ERBα to HSP90 (53) and the acetylation of GR by CLOCK (46), both leading to suppression of GR action.

The interaction between GR and members of the molecular clock and its influence on hepatic metabolism can be further exemplified focusing on a single molecular factor. REV-ERBα is one of the key transcriptional repressors in the molecular transcriptional clock, contributing to the characteristic circadian expression in many tissues, including the brain and metabolic tissues like the liver, muscle, pancreas and adipose tissue. In the liver, REV-ERBα is involved in the daily regulation of glucose and lipid metabolism (153). REV-ERBα represses clock genes by binding to RevDR2/RORE DNA elements and recruiting the corepressor complex NCoR-HDAC3. On the other hand, REV-ERBα regulates many metabolic genes by tethering to cell-type specific TFs. Hepatic REV-ERBα tethers to e.g., HNF6 and recruits HDAC3 for active repression of lipogenic genes (154, 155). GR has been found to interact with REV-ERBα on different levels. REV-ERBα interacts physically with GR and, together with HNF4α and HNF6, binds regulatory regions controlling gene expression in mouse liver. REV-ERBα was found to be important for efficient GR recruitment to chromatin during the day, presumably by maintaining histone acetylation at binding sites (82). Moreover, indirect interactions between GR and REV-ERBα have also been observed. REV-ERBα inhibits GR protein expression and nuclear localization (53), and GR inhibits REV-ERBα RNA expression (156) by forming a complex with CLOCK and BMAL1, where GR may be tethered to the regulatory site of the REV-ERBα gene (115).

## Perspectives in Disease and Clinical Use of GCs

Glucocorticoids have immunosuppressant and anti-inflammatory properties, making them an effective treatment for allergies, inflammatory and autoimmune diseases. The anti-inflammatory effects mediated by GR are conducted by the immune cells, with the macrophages having a particularly important role in the repression of inflammatory genes [reviewed in (157)]. However, by administering GCs systemically, there is a risk of eliciting undesirable side effects on other tissues and cellular processes, such as hepatic metabolism, which is highly impacted by GR regulation. In this review, we described the multiple layers of regulation of GR function, from the control of hormonal availability to the modulation of GR expression at both mRNA and protein levels, as well as PTMs and interactions with different proteins and TFs affecting the transcriptional activity of GR. In depth knowledge of the multifaced control of GR activity provides a unique opportunity to tailor GC treatment and prevent metabolic-related side effects.

One strategy could involve administration of different GR ligands affecting interacting coregulators to modulate transcriptional regulation by GR (8). Another strategy could be to selectively activate or inhibit specific and relevant GR-mediated regulatory pathways, where treatments involving a combination of different TF ligands could have potential. For example, co-administration of GC and LXR agonists attenuates the transcriptional activity of GR on a subset of genes in glucose and lipid metabolism, suggesting co-treatment with LXRβ agonists might reduce metabolic side effects in patients with autoimmune or inflammatory diseases (112). However, the function of LXRs on GR target sites is debated (113), and the mechanisms behind the positive and negative effects of LXRs on GR should be elucidated. Also, the antagonistic effect of activated PPARα on GR-mediated transcription of metabolic genes to circumvent GC side effects seems promising (150), with potentials and challenges recently discussed in another review (1). Additionally, the natural ultradian GC release and subsequent dynamic activation of GR contrasts with the constant exposure to GCs during pharmacological therapies. The development of new synthetic GCs and pulsatory administration strategies could potentially minimize side effects by mimicking physiology (58, 59, 103). Finally, pharmacological chronotherapy involving GCs seems promising in several inflammatory disorders, with outcomes improving when GC administration is consistently timed (4). This timed GC-administration has been shown to be beneficial in, for example, patients with rheumatoid arthritis (158).

## Concluding Remarks

The multifaceted regulation of GC action and GR activity discussed in this review highlights the complexity of transcriptional regulation by ligand-dependent TFs. The cooperation with signal-dependent and lineage-specific TFs makes GC-dependent gene regulation very responsive to environmental cues and is thus essential to understand for future optimized usage of GCs in the clinic. Specifically, a deeper understanding of the regulatory mechanisms underlying GR action would be fundamental for future development of safer and more effective therapies for disorders where GC secretion and signaling is involved. The recent genomics studies into the GR interactome show promise in the elucidation of the complex GR-TF networks and could contribute to a shift toward future tailored pharmacological strategies including spatio-temporal drug delivery and personalized medicine.

## Author Contributions

SP and CC wrote the manuscript with supervision from LG. All authors contributed to the article and approved the submitted version.

## Conflict of Interest

The authors declare that the research was conducted in the absence of any commercial or financial relationships that could be construed as a potential conflict of interest.
